# Pseudolymphomatous Granuloma Annulare: A Case Report

**DOI:** 10.3390/dermatopathology7020006

**Published:** 2020-09-08

**Authors:** Raphaël André, Yasmine Ibrahim, Aurélie Hsieh, Gürkan Kaya

**Affiliations:** 1 Department of Dermatology, University Hospital of Geneva, 1205 Geneva, Switzerland; yasminelucile.ibrahim@hcuge.ch (Y.I.); aurelie.hsieh@hcuge.ch (A.H.); guerkan.kaya@hcuge.ch (G.K.); 2 Department of Clinical Pathology, University Hospital of Geneva, 1205 Geneva, Switzerland

**Keywords:** granuloma annulare, pseudolymphomatous granuloma annulare

## Abstract

Granuloma annulare is an idiopathic granulomatous condition. Clinical variants of granuloma annulare include classical and localized, large erythematous patch, generalized, perforating, and subcutaneous/deep forms. Rarely, granuloma annulare shows a prominent lymphoid infiltration. This form is called pseudolymphomatous granuloma annulare. Here, we describe a new case of pseudolymphomatous granuloma annulare.

Granuloma annulare is an idiopathic granulomatous condition. Clinical variants of granuloma annulare include classical and localized, large erythematous patch, generalized, perforating, and subcutaneous/deep forms [1]. Histologically, granuloma annulare shows an infiltration of histiocytes and a perivascular infiltration of lymphocytes that is usually sparse. The histiocytes may be present in an interstitial pattern without apparent organization, or in palisades surrounding necrobiotic areas with prominent mucin. Rarely, granuloma annulare shows a prominent lymphoid infiltration. This form is called pseudolymphomatous granuloma annulare. Here, we describe a new case of pseudolymphomatous granuloma annulare.

## 1. Case Report

A 60-year-old woman presented to the outpatient clinic of the Department of Dermatology with a depigmentation of the neck for 3 months as well as pruritic elbow lesions. The elbow lesions appeared mainly during summer, and had been present for several years. This woman was originally from the Philippines and had no direct contact with tuberculosis. She had atopy, high blood pressure, and hypothyroidism since the resection of the thyroid gland for papillary carcinoma. Her treatment included levothyroxine, olmesartan, and vitamin D.

**Figure 1:**
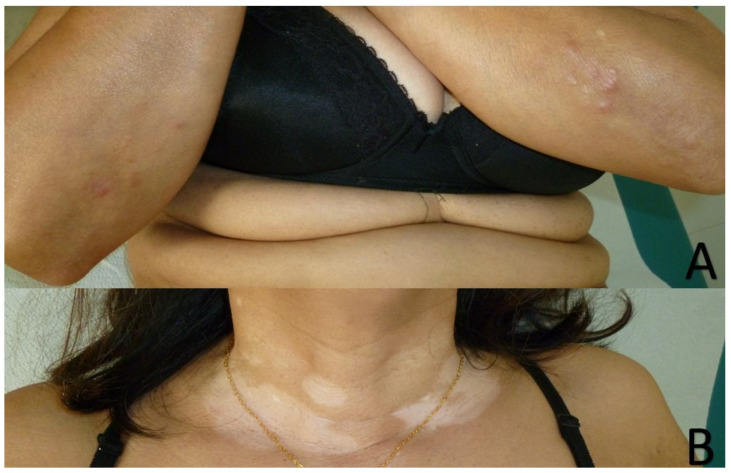
Erythematous, smooth, indurated papules on the extension side of the elbows (**A**). Vitiligo on the neck (**B**).

**Figure 2:**
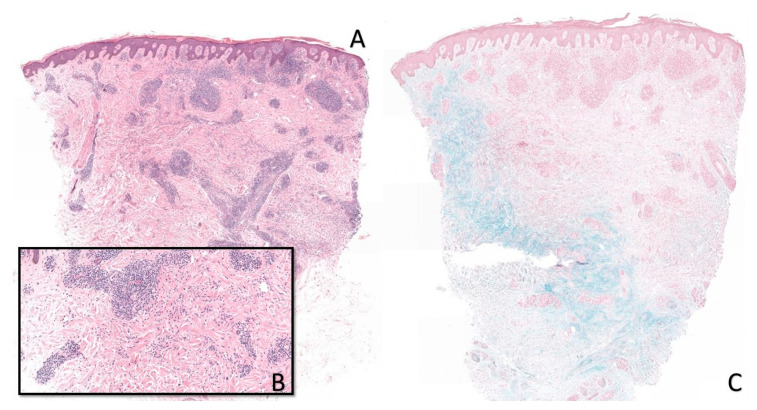
Pseudolymphomatous granuloma annulare. Dense perivascular and also interstitial lymphohistiocytic inflammation associated with a rare deep-extending eosinophilic infiltrate. There is no granuloma, and we can discern early palisading histiocytes (**A**, ×20). Detail of the lymphoid infiltrates and degenerative changes of the collagen (**B**, ×100). Mucin is prominent in the deep dermis (**C**, ×20).

Clinically, the depigmented neck lesions were consistent with vitiligo. On the extension side of the elbows, smooth, hard, and infiltrated erythematous papules were observed (Figure 1). In the presence of a wide differential diagnosis comprising granuloma annulare, sarcoidosis, interstitial granulomatous dermatitis, infection (leprosy and tuberculosis), and pseudolymphomatous arthropod bite reaction, a biopsy was performed. Histology showed a dense perivascular and interstitial lymphocytic infiltrate associated with focal histiocytic palisading around necrobiotic areas. Colloidal iron staining showed mucin deposition in the necrobiotic zones. Miller’s stain did not show elastophagocytosis. CD68 immunostaining showed the presence of histiocytes without palisade formation, but they were scattered and interstitial. These aspects were in favor of pseudolymphomatous granuloma annulare (Figure 2). There was no argument for an infectious origin on the different special stainings performed (Periodic Acid Schiff and Ziehl–Neelsen).

## 2. Discussion

Among the variants of granuloma annulare, the pseudolymphomatous form is very rare. It was described in 2012 by Cota et al. in 10 cases [2]. Since then, only one more case has been reported [3]. Among all of the reported cases, three patients had the same elbow location as our patient. Other locations included the lower limbs and trunk. One case had a generalized form. The pruritus was also described by Velasco et al. [3]. This clinical information was missing in the study by Cota et al. None of the cases was associated with cancer.

Histologically, our patient shared signs of conventional (necrobiotic) and interstitial granuloma annulare. This was found in 3/10 patients in the study by Cota et al. Six patients had a unique interstitial form and one patient had a unique conventional form. Eosinophils was not always present. Of the 11 cases reported in the literature, only two cases had eosinophils in the biopsy. Our patient’s histology also showed eosinophils. Based on data from the literature and from our patient, immunohistochemical analyses usually show a T lymphocyte infiltrate composed of CD4 and CD8 without atypia. The histiocytes are CD1a+, CD68+, and CD163+. CD123 is always negative.

The origin of granuloma annulare remains undetermined. A recent review has shown that three possible associations can be retained, namely: HIV infection, diabetes mellitus, and autoimmune hypothyroidism [4]. Drug-induced forms have also been reported (topiramate, allopurinol, Tumor necrosis factor-α inhibitors, or gold therapy). Our patient recently developed vitiligo. The association between granuloma annulare and autoimmune diseases (i.e., autoimmune hypothyroidism) has been described above. In addition, the association between vitiligo and autoimmune hypothyroidism is also known. In our patient, an autoimmune origin was therefore not excluded.

Finally, our patient described a flair-up during summer. This description is not usual, as phototherapy is known to have a slight beneficial effect on granuloma annulare [4]. This aspect was not described in the other reported cases. Only the description of other clinical cases will allow us to know whether this is a specific sign of the pseudolymphomatous form.
